# Heterogeneous Treatment Effects in the Incredible Years Teacher Classroom Management Programme - A Latent Profile Approach

**DOI:** 10.1007/s11121-024-01733-3

**Published:** 2024-09-24

**Authors:** Håvard Horndalen Tveit, Frode Stenseng

**Affiliations:** 1 The Regional Centre for Child and Youth Mental Health and Child Welfare – Central Norway, Norwegian University of Science and Technology (NTNU), Trondheim, Norway; 2 Department of Education and Lifelong Learning, Norwegian University of Science and Technology (NTNU), Paviljong B, 179, Dragvoll, Dragvoll Allé, Trondheim, Norway

**Keywords:** Treatment variability, Treatment interaction, Causal heterogeneity, Interindividual variation, Latent profile analysis

## Abstract

Heterogeneous effects from interventions often remain hidden in between-group analyses, risking overgeneralized conclusions of treatment effects. In this exploratory study, we performed latent profile analysis to unveil differential treatment effects among children in The Incredible Years Teacher Classroom Management Programme (IY TCMP). This program has previously been shown to reduce behavioral problems in preschools and schools in total samples and subgroups. A total of 726 children (48.7% girls; *M*_age_ = 4.21 years; *SD*_age_ = 0.86) from 92 childcare centers in Norway participated in either the intervention (n = 338) or the matched control condition (n = 388). First, by conducting latent profile analysis on baseline levels of child-teacher relationship (closeness, conflict), behavioral problems, and social competence, three distinct profiles were identified: *High Risk* (26.4%), *Moderate Risk* (42.8%), and *Low Risk* (30.7%) – each profile with unique characteristics. Second, we tested for within-profile, condition-by-time interactions following the intervention, showing distinct treatment responses for each profile. *High-risk* profiles profited most from the IY TCMP, with a substantial decrease in externalizing problems, more teacher closeness, and less teacher conflict. *Moderate-risk* profiles also gained better teacher-child relationships and improved social competence. The *Low-Risk* profiles showed no effects from the intervention. It is argued that latent profile analysis presents a feasible approach for examining within-sample heterogeneity in intervention research. It also reveals crucial information on treatment variability, as demonstrated in the Incredible Years Programme.

## Introduction

Human development is inherently heterogeneous – variations between individuals within a population are the cornerstone of evolution and an explicit premise in contemporary theories on individual development (Gottlieb, 2007, p. 431; Lerner, 1991). How some children prosper in their respective socio-relational contexts, whereas others develop problematic patterns of behavior and a vulnerability to emotional problems, is one of the focal areas of the developmental sciences (e.g., Belsky & Pluess, 2009). Thus, determining risk factors for such malfunctioning development, both within the child and from its proximal and distal contexts (Bronfenbrenner, 1979), is of crucial relevance for adapting governmental systems and practices that build psychological resilience in children in their institutions, such as preschools and schools (Masten, 2001).

Although the acknowledgment of individual variations across social contexts is widespread within the field of, e.g., child development, observed variations between participants in study samples are still commonly treated as measurement errors or chance deviations rather than reflecting true variations in the population (Bolger et al., 2019; Xie, 2013), this phenomenon is potentially leading to inaccurate or flawed conclusions about the data. The traditional statistical practice is intended to demonstrate general processes or characteristics at the population level, investigating the between-participant, or interindividual, variation (Fisher et al., 2018). Applying results from an aggregated sample response to an individual participant involves a shift from the between-participant to the within-participant level (Molenaar & Campbell, 2009), a shift only valid under conditions of strict within-population homogeneity, a condition rarely, if ever, met within the developmental sciences (Hamaker et al., 2005). In rationalizing natural variations within the population as mere statistical artifacts, there is a risk that models on human behavior and processes become irrelevant in their application (Nesselroade et al., 2007) – one size fits none.

### Acknowledging Heterogeneity

Assessing heterogeneity in early intervention research is imperative, as average treatment effects can conceal individual or subgroup variability. In extreme cases, subgroups may even score contrary to the aggregated sample response. Although statistical procedures for studying within-participants, or intraindividual, variation exists (Bolger et al., 2019; Hamaker et al., 2005), these approaches require significant amounts of data per participant, vastly surpassing any feasible data collection methods for most early interventions. An approximation to individual modeling of participants is to identify a set of behavioral markers predictive of the treatment response as a future reference for allocating participants into distinct treatment cohorts. By stratifying participants, one may locate participants experiencing harmful or null effects of an intervention, which, in addition to avoiding harm to participants, could provide incentives for allocating resources more effectively (Fröhlich et al., 2018). Exploring heterogeneity in the treatment response may give clues to the causal processes of an intervention (Hopwood, 2007) and lay the foundation for more efficient implementation (Ng & Weisz, 2016).

In line with the US National Institute of Mental Health’s (2020) call towards stratified interventions in mental health, the objective of the current paper is to investigate whether distinct groups of participants will achieve differential treatment effects of a prominent childcare and school intervention, namely the Incredible Years Teacher Classroom Management (IY TCM) programme (Webster-Stratton et al., 2008; Webster-Stratton, 2011), using Latent profile analysis (LPA) on a large data set of Norwegian preschool children participating in the IY TCM-intervention.

### The Incredible Years Programme

The Incredible Years Programme (Webster-Stratton et al., 2001; 2008; Webster-Stratton, 2011) is a series of interlocking, evidence-based programs for parents, children, and teachers. It aims to promote emotional and social competence and prevent, reduce, and treat behavior and emotional problems in young children. Different versions of the program have been developed for teachers, educators, and caregivers, typically aimed at children ages 3 to 8. The Incredible Years Teacher Management Programme is based on Patterson’s coercion theory (Patterson et al., 2002), attachment theory (Bowlby, 1969), and social-learning theory (Bandura, 1977), stipulating that the child’s interactions and relationships with its primary caregivers and other adults, such as teachers, affect the behavioral and emotional development of the child. For instance, because teacher’s confidence in their own educational and caregiving abilities is associated with positive child-teacher relationships, such as fewer conflictual interactions, the intervention is partly aimed at increasing teacher self-efficacy (Bandura, 2001). To achieve this objective, including others found to be favorable for the teacher-child relationship, teachers engage in guided workshops, with discussions, role-plays, video vignettes, and reading assignments (Webster-Stratton et al., 2011). Numerous studies have shown favorable changes in child and teacher-related outcomes following the IY TCM intervention (e.g. Pidano & Allen, 2015; Reinke et al., 2018; Tveit et al., 2020; Webster-Stratton et al., 2011); with reasonably consistent results across countries and contexts (for a review, see Nye et al., 2019). Typically, in previous IY TCM studies, results are presented as the average treatment effect of the programme, alongside effect size estimates, typically revealing small to moderate treatment magnitudes. However, in some studies, theoretically rationalized subgroups are included, e.g., children scoring above a given percentile on a given measure, demonstrating larger effect sizes than the general sample (see Reinke et al., 2018).

Although studies on rationalized subgroups may reveal potential markers of treatment response, subgroup analysis is often methodologically problematic, plagued with high Type I error rates, low statistical power, and usually interpreted with skepticism (Rothwell, 2005). An alternative approach to the traditional subgroup analysis, and a potential source of information on differential treatment responses, is using finite mixture modeling techniques.

### Latent Profile Analysis

Latent profile analysis (LPA) is a mixture modeling technique applied for continuous input variables, partitioning the sample into mutually exclusive and exhaustive latent cohorts (Lanza & Rhoades, 2013). LPA is oft-used when the researcher assumes that the within-sample heterogeneity is caused by an unmeasured, categorical grouping variable, attempting to form latent, homogenous cohorts based on the means and (co)variances of a set of indicators (Oberski, 2016; Peugh & Fan, 2013). The benefit of LPA over competing clustering techniques (e.g., k-means or hierarchical clustering) is that the LPA provides objective measures to evaluate the fit of a model while offering flexibility in model configurations (Scrucca et al., 2016). In intervention research, LPA is a sensible procedure for identifying subgroups of participants when applied to potential behavioral or demographical indicators of treatment variability, significantly outperforming the traditional subgroup approach.

The LPA approach has been applied in some recent studies using teacher-targeted intervention programmes (Hennessey & Humphrey, 2020; Zhao & Jin, 2023), but studies combining LPA with teacher outcomes in the IY Programme is still lacking. Thus, we here present two known studies combining LPA with the parent-targeted version of the IY Programme. Weeland et al. (2023) identified parenting profiles through LPA in a large sample of parents (785 caregiver-child dyads) participating in the Incredible Years Parenting Programme. They identified three profiles of parenting involvement based on baseline assessments: *High Involvement* (8.4%), *Low Involvement* (81.4%), and *Harsh Parenting* (10.1%). These profiles were used to determine the variability of treatment effects in the program. However, contrary to expectations, changes in these profiles were not observed following the intervention, nor were differential outcomes on child behavior observed based on these profiles. Thompson et al. (2017) used LPA to identify four groups of parental involvement in a child’s school functioning based on teacher reports. They found that parents with a profile of low school involvement were rated more favorably by the same teachers after the intervention.

To our knowledge, no studies have used LPA to identify risk profiles among children, followed by analyses of differential treatment effects from the IY TCM, to determine the generalizability, or the lack of such, relating the anticipated impact on child behavior from the program.

### The Present Study

The main objective of the present study was to explore whether latent profiles based on shared baseline characteristics would demonstrate differential treatment effects following the IY TCM intervention. Using data from a quasi-experimental pre-post trial with a matched control condition of the IY TCM performed in Norwegian childcare centers, we applied LPA to identify latent subgroup profiles by using the target outcomes as predictors, i.e., behavioral problems, emotional problems, and social competence. Next, we investigated the differences between these identified subgroups at the baseline assessment. Finally, to explore treatment variability from the IYT CM intervention, a series of hierarchical linear mixed models of the condition-by-time interaction were run for each latent profile.

## Method

### Trial Procedures

The Regional Committee for Medical and Health Research Ethics approved the trial protocol and intervention procedures, North of Norway (ref. 2009/655/REK NORTH) before the trial began. The teacher and parents were informed of the trial objectives and intervention procedures and of their right to refuse participation or withdraw their consent at any time without reprisal, as stated in the Helsinki Declaration. All data were anonymized in accordance with national regulations.

The trial objective was to implement the IY TCM programme as a universal intervention in a representative sample of Norwegian childcare centers, and to study the hypothesized changes following the intervention in the child-teacher relationship, as well as the children’s emotional and behavioral problems, and social competence. The trial employed a quasi-experimental pre-post design with a matched control condition, matched on geographical context (rural versus urban) and centers size, as these were the potential main confounders in a relatively homogeneous population.

To minimize potential conflicts of interest, the Norwegian Directorate of Health, as the founder of the study, called for strict boundaries between the national IY agency, facilitating the implementation, and the researchers set to evaluate the intervention. The IY agency followed its protocols for ensuring program fidelity and teacher participation. At the same time, the research group was responsible for the trial protocol, design of the trial, data collection, analyses, and recruitment of childcare centers.

### Sample Recruitment

To be eligible for participation in the intervention condition, all units at the childcare center had to participate, 80% of the childcare teachers had to approve of participation, and the teachers were not allowed to participate in any other behavioral or educational training programs during the intervention period. The teachers were further instructed only to use the behavioral strategies they learned during the IY TCMP workshops. If accepted, the childcare center would receive the IY TCM programme free of charge and a modest compensation for the time spent completing the questionnaires. Childcare centers in the control condition were accepted, provided the specified matching criteria were met. Childcare centers in the control received the same compensation for completing the questionnaires and received the IY TCM intervention free of charge the following year.

The sample size estimation was based on detecting an effect size of 0.20 with a 0.05 level of significance and 0.80 level of power for a two-sided test. As participants were nested within units, the design effect was estimated as the product of the intraclass correlation coefficient and the mean cluster size. Following a randomized selection process, seven children from each unit were chosen to participate in the assessment, resulting in a total of 581 children in the intervention and 637 in the control condition.

### Participants

A total of 1218 children (49.7% girls; *M*_*age*_ = 4.22 years; *SD*_*age*_ = 0.88) were included in the study: 581 children (51.1% girls; *M*_*age*_ = 4.22 years; *SD*_*age*_ = 0.88) in the intervention condition and 637 children (48.3% girls; *M*_*age*_ = 4.27 years; *SD*_*age*_ = 0.85) in the control. 90.4% of the children spent 30 h or more per week at the childcare centre, approximately the same as the national average of 91.8% (Statistics Norway, 2021). Forty-seven children had special educational needs, similar to the 4.2% national average. Ninety-two childcare centers were included in the study, representing 8 out of 11 counties in Norway, with an equal share of private and municipal ownership. The average group size in both conditions was 19 children, and 9.2% of the childcare staff were males, similar to the national average (Statistics Norway, 2021). A total of 92.8% of all children in Norway attend childcare, and a total of 19% of all children belong to linguistic and cultural minorities.

### Intervention Procedures

The IY TCM programme is comprised of six full-day workshops, held approximately every fourth week over nine months, and includes the entire childcare staff. The implementation was organized by two IY TCM-certified group leaders, and followed the procedures as described in the manual (Webster-Stratton, 2011), with a focus on promoting strict programme fidelity. In brief, the workshops intend to promote childcare teacher’s confidence and self-efficacy in forming meaningful child-teacher relationships, emphasizing a learning-by-doing approach, including role-play, video-vignettes, self-reflection, and discussion with sharing of experiences. More specifically, the topics of the workshops were: (i) Building a positive relationship with the child and preventing behavior problems; (ii) The importance of teacher attention and praise; (iii) Motivating children through incentives; (iv) Ignoring and redirecting inappropriate behavior; (v) Following through with consequences; (vi) Developing the children’s emotional regulation, social skills, and problem-solving (Webster-Stratton, 2011). In addition, the childcare teachers receive reading assignments, one-on-one mentoring, and are to practice the IY TCM principles when back at their units.

### Measures

*Child-Teacher Relationship*. The Student-Teacher Relationship Scale, Short Form (STRS; Pianta, 2001) is a self-report questionnaire for teachers, which consists of 15 items, rated on a 5-point Likert scale, assessing the teacher's perception of their relationship to a particular child. The STRS includes the closeness dimension (eight items; α = 0.80 at baseline), measuring the level of warmth and affection in the relationship, and the conflict dimension (seven items; α = 0.80 at baseline), measuring the level of coercion and discord.

*Behavioral Problems.* The Caregiver-Teacher Report Form, 1.5–5 (CTRF; Achenbach & Rescorla, 2000) consists of 99 items, rated on a 3-point Likert scale, assessing the children’s level of behavioral problems. The CTRF includes the internalizing problems scale (32 items; α = 0.85 at baseline), measuring the degree of emotional problems, and the externalizing problems scale (34 items; α = 0.94 at baseline), measuring the degree of attention problems and aggressive behavior.

*Social Competence*. The Social Competence and Behavior Evaluation for Teachers (LaFreniere & Dumas, 1995) consists of 40 items rated on a 6-point Likert scale, assessing the child’s interactional skills and prosocial behavior. Cronbach’s alpha for the SCBE was 0.97 at baseline.

### Statistical Analyses

All analyses were conducted in *R* (R Core Team, 2019) version 3.6.0. The latent profile analysis was run with the *mclust* package (Scrucca et al., 2016), the *rstatix* package (Kassambara, 2020) was used to test for interaction effects, and the linear mixed models was conducted with a combination of the *lme4* (Bates et al., 2015) and the *lmerTest* package (Kuznetsova et al., 2017). The visualization was done with *ggplot2* (Wickham, 2016).

The standard procedure for dealing with missing items outlined in all three manuals was identical – granted the number of missing items was below a given threshold, mean imputation within-participant and subscale were recommended, while if the number of missing items was above the threshold, the scale was left as missing. The respective thresholds are one missing item per subscale of the STRS (Pianta, 2001), eight of the total 99 items of the CTRF (Achenbach & Rescorla, 2000), and four items of the Social Competence Scale (LaFreniere & Dumas, 1995).

The latent profile analysis was estimated as a Gaussian finite mixture model running the Expectation Maximisation algorithm on the following pre-test predictors of emotional problems, behavioral problems, and social competence. In specifying profile attributes, the default setting in the mclust-package was used, allowing for up to nine profiles and a total of 14 model variants to be tested. The Bayesian Information Criterion (BIC) was used in determining the optimal profile and variant configuration (Fraley & Raftery, 1998), retaining the model with a BIC-value closest to zero.

To examine the latent profiles, a series of between-subjects ANOVAs was conducted to investigate their effects on each of the five baseline outcome scales. In the case of a statistically significant difference, the ANOVA was followed by Tukey’s HSD test for multiple pairwise comparisons between profiles.

Finally, a series of linear mixed models were run to investigate the condition-by-time interaction on each of the outcome measures individually for each latent profile, the repeated measures (level 1) nested within children (level 2) nested within teachers (level 3). The mixed models were run with full information maximum likelihood estimation, with the results reported as the estimated marginal means and the standard error from the model, and the effect size computed as Hedges’ *g*.

## Results

The effective sample consisted of 726 children (48.7% girls; *M*_age_ = 4.21 years; *SD*_*age*_ = 0.86), 338 children (48.8% girls; *M*_age_ = 4.19; *SD*_*age*_ = 0.87) in the intervention condition, and 388 children (48.7% girls; *M*_age_ = 4.24; *SD*_*age*_ = 0.87) in the control condition. A total of 90.2% of the children (91.4% in the intervention and 89.2% in the control) spent 30h or more per week at the childcare centre.

**Fig. 1:**
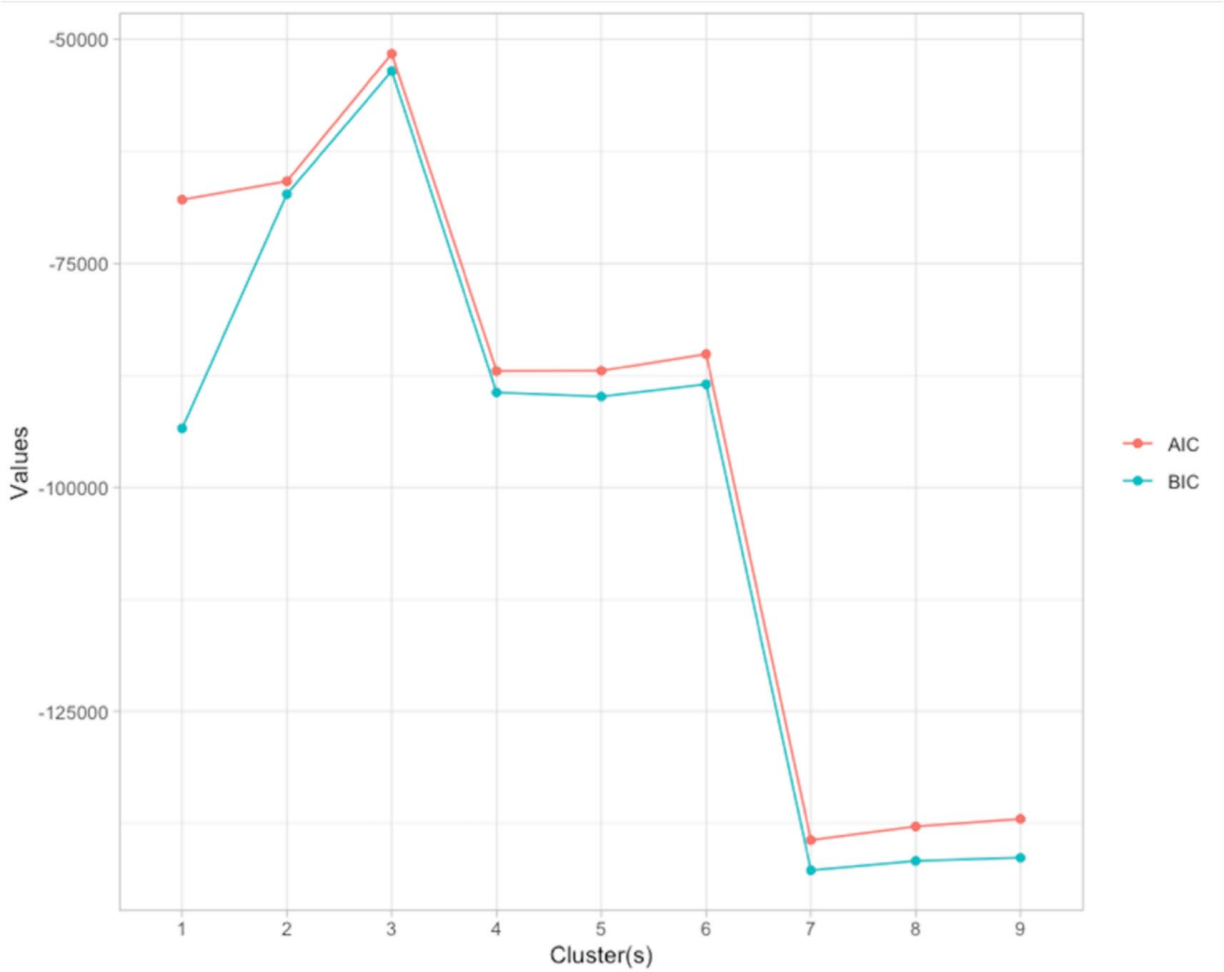
Elbow-plot of AIC and BIC indices for the latent profile analysis. Note. The ICL values converged with the AIC values to the extent that they were redundant in the plot

**Fig. 2:**
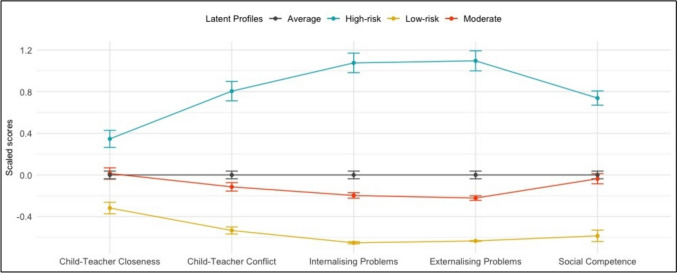
Standardized baseline scores for the three latent profiles on the outcome measures. *Note.* Child-Teacher Closeness and Social Competence scores are reversed for illustrative purposes

The latent profile analysis identified the best fitted solutions to be three profiles (BIC = -53,538; ICL = -53,541; AIC = -51,612), two profiles (BIC = -67,276; ICL = -67,277; AIC = -65,836) or six profiles (BIC = -88,497; ICL = -88,533; AIC = -85,134), with the optimal solution based on the BIC, ICL and elbow plot to be the three profile solution (see Fig. 1). Following the three profile solution, Profile 1 (*N*_*intervention*_ = 102 [30.2%]; *N*_*control*_ = 121 [31.2%]) was labeled *Low-risk*, scoring most favorable on every outcome measure; Profile 2 (*N*_*intervention*_ = 147 [43.5%]; *N*_*control*_ = 164 [42.3%]) was labeled *Moderate*, scoring closest to the average sample score; while Profile 3 *(N*_*intervention*_ = 89 [26.3%]; *N*_*control*_ = 103 [26.5%]) was labeled *High-risk*, characterized by the highest scores on all outcome measures (see Fig. 2).

The between-subjects ANOVAs revealed a significant baseline difference between the profiles on Child-Teacher Closeness (*F*[2, 723] = 24.283, *p* < 0.001, η^2^_G_ = 0.063), Child-Teacher Conflict (*F*[2, 723] = 130.095, *p* < 0.001, η^2^_G_ = 0.265), Internalizing Problems (*F*[2, 723] = 300.437, *p* < 0.001, η^2^_G_ = 0.454), Externalizing Problems (*F*[2, 723] = 311.558, *p* < 0.001, η^2^_G_ = 0.463), and Social Competence (*F*[2, 723] = 120.601, *p* < 0.001, η^2^_G_ = 0.250). Tukey’s HSD Tests for multiple pairwise comparisons were statistically significant (*p* < 0.001) for all pairwise profile combinations, which, along with the respective effect sizes, demonstrates the difference between the profiles at the baseline.

The linear mixed models showed no statistically significant baseline differences between the intervention and control condition within any of the profiles (Table 1). Testing the condition-by-time interaction of the *Low-risk* profile, showed that the intervention and the control condition did not change significantly different on any outcome measures. For the *Moderate-risk* profile, children in the intervention condition changed more favorably than children in the control condition on the Child-Teacher Closeness (1.3 index points; 95% CI [0.57, 2.03]; *p* = 0.001; *g* = 0.19), Child-Teacher Conflict (1.0 index point; 95% CI [0.14, 1.86]; *p* = 0.024; *g* = 0.15), and Social Competence (6.3 index points; 95% CI [0.98, 11.63]; *p* = 0.021; *g* = 0.11), but not on Internalizing Problems (0.28 index points; 95% CI [–0.94, 0.38]; *p* = 0.404) or Externalizing Problems (0.99 index points; 95% CI [–2.01, 0.04]; *p* = 0.061). Finally, children classified in the *High-risk* profile demonstrated significant condition-by-time changes in favor of the intervention on Child-Teacher Closeness (2.11 index points; 95% CI [1.06, 3.16]; *p* = 0.001; *g* = 0.23), Child-Teacher Conflict (1.81 index point; 95% CI [0.44, 3.19]; *p* = 0.01; *g* = 0.13), and Externalizing Problems (2.65 index points; 95% CI [0.52, 4.78]; *p* = 0.016; *g* = 0.12), though not on Internalizing Problems (1.14 index points; 95% CI [–2.49, 0.21]; *p* = 0.1) or Social Competence (6.53 index points; 95% CI [–0.85, 13.91]; *p* = 0.085).

**Table 1:** Estimated marginal means and standard errors at baseline and follow-up

	Control Condition		Intervention Condition		Differences between conditions				
	Baseline	Follow-up	Baseline	Follow-up	Baseline differences		Condition-by-time interaction		
	Mean ± *SE*	Mean ± *SE*	Mean ± *SE*	Mean ± *SE*	Estimate [95% CI]	*p*	Estimate [95% CI]	*p*	*g*
**Closeness**									
Low-risk	31.4 ± 0.4	31.7 ± 0.4	30.6 ± 0.4	31.4 ± 0.4	0.82 [–0.23, 1.87]	0.130	0.54 [–0.42, 1.50]	0.274	0.07
Moderate	30.3 ± 0.4	30.9 ± 0.4	29.4 ± 0.4	31.3 ± 0.4	0.85 [–0.15, 1.86]	0.099	1.30 [ 0.57, 2.03]	0.001	0.19
High-risk	28.7 ± 0.5	29.3 ± 0.5	28.3 ± 0.5	31.0 ± 0.5	0.43 [–0.92, 1.79]	0.533	2.11 [ 1.06, 3.16]	0.001	0.23
**Conflict**									
Low-risk	9.6 ± 0.3	10.1 ± 0.3	9.8 ± 0.3	9.8 ± 0.3	0.16 [–0.66, 0.98]	0.696	–0.51 [–1.35, 0.32]	0.230	0.09
Moderate	11.6 ± 0.3	12.0 ± 0.3	11.6 ± 0.4	11.0 ± 0.4	0.01 [–0.98, 1.00]	0.985	–1.00 [–1.86, –0.14]	0.024	0.15
High-risk	15.4 ± 0.7	15.5 ± 0.7	16.8 ± 0.8	15.1 ± 0.8	1.37 [–0.66, 3.40]	0.188	–1.81 [–3.19, –0.44]	0.010	0.13
**Internalizing**									
Low-risk	0.4 ± 0.1	1.2 ± 0.1	0.4 ± 0.1	1.0 ± 0.1	0.03 [–0.33, 0.39]	0.886	–0.17 [–0.59, 0.26]	0.435	0.07
Moderate	2.1 ± 0.2	2.3 ± 0.2	2.3 ± 0.3	2.2 ± 0.3	0.19 [–0.48, 0.87]	0.573	–0.28 [–0.94, 0.38]	0.404	0.06
High-risk	7.4 ± 0.5	6.3 ± 0.5	7.3 ± 0.5	5.0 ± 0.6	0.15 [–1.30, 1.59]	0.844	–1.14 [–2.49, 0.21]	0.100	0.11
**Externalizing**									
Low-risk	0.3 ± 0.2	1.0 ± 0.2	0.3 ± 0.2	1.1 ± 0.2	0.02 [–0.44, 0.49]	0.918	0.07 [–0.51, 0.65]	0.809	0.02
Moderate	3.5 ± 0.3	3.7 ± 0.3	3.6 ± 0.4	2.9 ± 0.4	0.12 [–0.83, 1.07]	0.810	–0.99 [–2.01, 0.04]	0.061	0.15
High-risk	13.2 ± 1.1	11.5 ± 1.1	15.1 ± 1.2	10.8 ± 1.2	1.95 [–1.25, 5.15]	0.236	–2.65 [–4.78, –0.52]	0.016	0.12
**Social Competence**									
Low-risk	193.0 ± 3.2	196.9 ± 3.2	189.5 ± 3.5	197.2 ± 3.6	3.43 [–5.93, 12.79]	0.474	3.81 [–1,29, 8.90]	0.145	0.06
Moderate	178.8 ± 2.8	183.8 ± 2.8	170.8 ± 3.0	182.1 ± 3.0	7.99 [–0.04, 16.02]	0.053	6.30 [ 0.98, 11,63]	0.021	0.11
High-risk	154.0 ± 3.3	163.0 ± 3.4	147.1 ± 3.5	162.7 ± 3.6	6.91 [–2.62, 16.45]	0.158	6.53 [–0.85, 13.91]	0.085	0.10

Results are reported as the estimated marginal means with standard errors from the linear mixed models. The effect size was computed as the estimate of the group-by-time effect divided by the pooled standard deviation at baseline

## Discussion

Individual variability in children's interaction with their social-relational contexts is highly acknowledged in developmental theories (e.g., Belsky & Pluess; Masten, 2001). However, such heterogeneity is often overlooked when determining intervention treatment effects (Bolger et al., 2019). This is not surprising because the main effects on the highest level of generalizability are what most interventions are targeting. However, this approach may mask high variability in treatment effects among participants (Lanza & Rhoades, 2013). In the current study, we explored the potential of using latent profile analysis as a means of unveiling differential treatment responses in the Incredible Years Teacher Classroom Management Programme (Webster-Stratton et al., 2001; 2008; Webster-Stratton, 2011), an intervention program shown to reduce behavioral and emotional difficulties among children aged 3 to 9 years (Pidano & Allen, 2015; Reinke et al., 2018; Tveit et al., 2020; Webster-Stratton et al., 2011). More specifically, we investigated the condition-by-time interaction within latent profiles following the delivery of the IYT PM preschools. These profiles were identified from baseline measures of child-teacher relationships, the children’s internalizing and externalizing behavior problems, and the children’s social competence. Three profiles were identified: a *Low-risk* profile scoring the most favorably on all measures, which included about one fourth of the sample, a *Moderate-risk* profile with about half of the sample, scoring about average on all measures, and a *High-risk* profile scoring the least favorably. Furthermore, in testing for the condition-by-time interaction within each distinct latent cluster, the three profiles showed differential treatment responses on several outcome measures – a probable proxy of treatment variability. In other words, substantial treatment variability from the IYT PM was found in the sample based on these analyses.

Specifically, children in the *High-risk* subgroup changed most favorably on child-teacher relationship quality, with more closeness and less conflict after the intervention. Additionally, they were reported to project fewer externalizing problems by teachers. The *Moderate-risk* subgroup displayed similar effects on child-teacher relationships, but in addition, it also projected improved social competence. The *Low-risk* subgroup was not reported to benefit from the intervention on any outcome measures controlled for baseline levels. In other words, the IY TMP was reported to have favorable effects for almost 70% of the sample. This illustrates the program’s broader relevance for preschool children through its effectiveness not just on children with severe behavior problems but also on those with no explicitly displayed problems. Interestingly, among those children projecting such explicit problems, the IY TPM did seem to facilitate improved social competence. Acknowledging that social competence is the cornerstone of many behavioral problems (Hukkelberg et al., 2019), as well as being highlighted as a resilience factor in emotional problems in middle childhood and adolescence, the present findings indicate that the program may have long-term preventive effects in a more general “well-being” perspective for larger parts of the population (Holopainen et al., 2012).

Of methodological relevance, the current findings demonstrate how latent profile analysis may be conducive when investigating differential treatment effects in intervention research. This has been pointed out as a pivotal task when evaluating interventions carried out in larger populations (Ng & Weisz, 2016). Heterogeneity was evidenced by the three distinct profiles emerging from the baseline analyses, while the differential treatment responses further supported the profiles' ecological validity. To that end, the present results support a diverse approach to intervention research among children, in line with the US National Institute of Mental Health (2020) recommendations. Furthermore, the findings call into question Rose's population strategy for prevention, which promoted the universal as superior to the high-risk approach to intervention, arguing that "a large number of people at a small risk may give rise to more cases of disease than the small number who are at a high risk" (Rose, 2001, p. 431). However, this proposition only holds true provided that universal treatment affects the entire population to some extent, which is a perhaps questionable proposition, as shown by the non-significant treatment effects in the *Low-risk* profile in the current study. In fact, the IY TCM programme was initially intended as a program for children with severe behavioral difficulties (Webster-Stratton, 1984), and not as a universal measure. This approach may be challenged following the advent of more sophisticated means towards identifying high-risk individuals, with the added benefit of allocating resources where needed most, rather than towards projected null effects. Overall, on the community level, the costs and benefits involved in delivering tailored programs to broader populations must be interrogated carefully (Charles et al., 2013).

Notably, in contrast to the traditional subgroup analysis, where indicator variables are selected based on theoretically rationalized markers of variation, the latent profile approach is empirically driven from the data at hand, which may thus reveal patterns unbeknownst to the researcher. Typically, in subgroup analyses, investigators divide the study sample into dichotomous groups based on one or more indicator variables, such as age, socioeconomic status, or some risk-assessment markers. This procedure becomes progressively more complex per included indicator; using only the five sum-score measures included in this study would result in 2^5^ = 32 subgroups. This type of dichotomization will lead to small, often minuscule, subgroups suffering from low statistical power, reducing the probability of detecting a true effect (Rothwell, 2005), while applying multiple tests for differences between subgroups will inflate the likelihood of Type I errors, resulting in a high probability of false positives. By applying the latent profile approach, however, the within-sample heterogeneity is regarded as a potential source of treatment variability, thus laying the groundwork for more meaningful and accurate interpretations (Lanza & Rhoades, 2013). Notably, the LPA approach may also be fruitful in determining differential cross-cultural effects of intervention programs such as the IY TCM Programme, which has been indicated in a comprehensive mixed methods review (Nye et al., 2019). The cost-effectiveness of the IY TCM has also been interrogated, with the conclusion of a beneficial effect, at least over a 9-month interval, but questioning effects over longer intervals, such as 18 and 30 months (Ford et al., 2019). More detailed analyses using LPA may reveal withstanding long-term effects among some clusters of children compared to others. Knowledge of what works best for whom and when may guide professionals in targeting those children which benefits the most, and this does not only count for the IY Programmes but also other comparable programs (see Hennessey & Humphrey, 2020; Zhao & Jin, 2023). As such, governmental aims of building resilience among children who are most vulnerable to adversities may be informed by studies using a more differential approach, such as the present one.

Latent profile analysis is not without its limitations. As with most exploratory, data-driven techniques, the analysis may best be understood as a procedure for identifying the best-fitted model, with a disregard to theory. Thus, spurious and nonsensical clusters may occur, and the latent profile analysis ought to be followed by critical analyses and attempts at validation. It is also important to recognize that every individual is given a probability of belonging to any of the latent profiles and that the individual is assigned to the profile with the highest probability. Yet, in an ill-fitting model, the probability of belonging to profile A, in contrast to profile B, may come down to the decimals (Muthén & Muthén, 2000). Thus, the profiles identified in this study may not be replicable, nor must they be interpreted as necessarily representing natural subgroups in the population: the objective was to explore how the distinct characteristics of the children at baseline may result in differential treatment effects. Also, due to being an intervention study carried out in naturalistic settings, it is conceivable that episodic effects, uncontrolled in the analyses, may have occurred during the study, affecting the outcomes. Finally, it should be noted that the child-teacher relationship scale is a self-report questionnaire and thus risks biasing parts of the analyses.

To summarize, using latent profile analysis in intervention research has the potential for elucidating treatment variability, thus bringing the field of intervention research closer to a true representation of individual variation in treatment effects. In the sequential process outlined herein, using data from a universal one-year implementation of The Incredible Years Teacher Management Programme (Webster-Stratton et al., 2001; 2008; Webster-Stratton, 2011) in Norwegian preschools, latent socio-behavioral markers were identified at baseline to create subgroups and differential effects of the program on the respective subgroups were identified at follow-up. Findings illustrate that tailored early interventions may be feasible, and more easily obtained than many believe, through applying latent profile analyses. As such, the current study exemplifies how refined statistical approaches may lead to more efficient use of resources in facilitating better socio-behavioral development among preschool children.
